# Aucubin promotes bone-fracture healing via the dual effects of anti-oxidative damage and enhancing osteoblastogenesis of hBM-MSCs

**DOI:** 10.1186/s13287-022-03125-2

**Published:** 2022-08-19

**Authors:** Kanbin Wang, Chengwei Zhou, Lijun Li, Chengxin Dai, Zhongxiang Wang, Weijun Zhang, Jianxiang Xu, Yueliang Zhu, Zhijun Pan

**Affiliations:** 1 Department of Orthopedic Surgery, The Fourth Affiliated Hospital, International Institutes of Medicine, Zhejiang University School of Medicine, No. N1 Shangcheng Road, Yiwu, 322000 People’s Republic of China; 2 Department of Orthopedic Surgery, The Second Affiliated Hospital, Zhejiang University School of Medicine, No. 88, Jiefang Road, Hangzhou, 310009 People’s Republic of China

**Keywords:** Aucubin, hBM-MSCs, Osteoblastogenesis, Anti-oxidative stress

## Abstract

**Background:**

Aucubin (AU), an iridoid glucoside isolated from many traditional herbal medicines, has anti-osteoporosis and anti-apoptosis bioactivities. However, the effect of AU on the treatment of bone-fracture remains unknown. In the present study, the aims were to investigate the roles and mechanisms of AU not only on osteoblastogenesis of human bone marrow-derived mesenchymal stromal cells (hBM-MSCs) and anti-oxidative stress injury in vitro, but also on bone-fracture regeneration by a rat tibial fracture model in vivo.

**Methods:**

CCK-8 assay was used to assess the effect of AU on the viability and proliferation of hBM-MSCs. The expression of specific genes and proteins on osteogenesis, apoptosis and signaling pathways was measured by qRT-PCR, western blotting and immunofluorescence analysis. ALP staining and quantitative analysis were performed to evaluate ALP activity. ARS and quantitative analysis were performed to evaluate calcium deposition. DCFH-DA staining was used to assess the level of reactive oxygen species (ROS). A rat tibial fracture model was established to validate the therapeutic effect of AU in vivo. Micro-CT with quantitative analysis and histological evaluation were used to assess the therapeutic effect of AU locally injection at the fracture site.

**Results:**

Our results revealed that AU did not affect the viability and proliferation of hBM-MSCs. Compared with control group, western blotting, PCR, ALP activity and calcium deposition proved that AU-treated groups promoted osteogenesis of hBM-MSCs. The ratio of phospho-Smad1/5/9 to total Smad also significantly increased after treatment of AU. AU-induced expression of BMP2 signaling target genes BMP2 and p-Smad1/5/9 as well as of osteogenic markers COL1A1 and RUNX2 was downregulated after treating with noggin and LDN193189. Furthermore, AU promoted the translocation of Nrf2 from cytoplasm to nucleus and the expression level of HO1 and NQO1 after oxidative damage. In a rat tibial fracture model, local injection of AU promoted bone regeneration.

**Conclusions:**

Our study demonstrates the dual effects of AU in not only promoting bone-fracture healing by regulating osteogenesis of hBM-MSCs partly via canonical BMP2/Smads signaling pathway but also suppressing oxidative stress damage partly via Nrf2/HO1 signaling pathway.

## Introduction

Fracture nonunion, a serious complication of bone-fracture regeneration and repair, remains one of the troublesome challenges leading to secondary revision surgery [1–3]. It is reported that the probability of fracture nonunion is about 1.9–9% [4], which is mainly dominated by mechanical instability of the fracture region [5]. In addition, systemic disease, infection, blood supply of fracture site and oxidative stress damage are the important factors of fracture delayed or nonunion [2, 6]. Recently, many studies paid attention to oxidative stress damage induced by ROS accumulation [7–10]. Oxidative stress microenvironment at the fracture site, caused by excessive accumulation of ROS, leads the osteoblasts to lose their biological function of self-renewal, proliferation and differentiation, and then affecting the bone-fracture healing process [11, 12]. Thus, the improvement in the resistance of cells and tissues to oxidative stress damage and reduction in ROS levels are essential for bone-fracture regeneration.

Bone marrow-derived mesenchymal stromal cells (BM-MSCs), with self-replication capabilities and multi-lineage differentiation potentials [13–15], play a key role in bone-fracture repair and regeneration. In the presence of bone injury signals, BM-MSCs are regulated by a variety of signaling factors to recruit and migrate to provisional callus, eventually leading to bone-fracture regeneration through endochondral and intramembranous ossification [16, 17].

Aucubin (AU), an iridoid glucoside, can be extracted from many traditional herbal medicines [18]. Several lines of evidence suggest that AU has a wide range of pharmacological properties including antioxidation, anti-aging, anti-inflammation, anti-tumor, anti-osteoporosis and hepatoprotection [18, 19]. Recently, its anti-oxidative stress and anti-osteoporosis functions in bone metabolism have attracted much attention [20, 21]. Nevertheless, few studies mentioned the role of AU on bone-fracture healing and the underlying mechanisms remain unknown. In this study, we explored the dual effects of AU on bone-fracture regeneration by its capability of anti-oxidative damage and promotion of the osteogenesis of hBM-MSCs.

## Materials and methods

### Cell culture and antibodies

hBM-MSCs that can be induced to differentiate into osteoblasts, chondrocytes and adipocytes under certain conditions are provided by Cyagen Biosciences (Guangzhou, China). The cells were cultured in hBM-MSC growth medium (Cyagen Biosciences, Guangzhou, China) at 37 °C in a cell incubator containing 5% CO_2_. The medium was refreshed every 3 days. hBM-MSCs were passaged at around 80–90% confluence, and then only passages three to seven were used in the subsequent experiments.

Aucubin (cat# HY-N0664), LDN193189 (cat# HY-12071) and Noggin (cat# HY-P70558) were purchased from MedChemExpress (Shanghai, China) and stored at − 80 °C after being dissolved in dimethyl sulfoxide (DMSO). NE-PER nuclear and cytoplasmic extraction reagents (cat# 78833) were purchased from Thermo Scientific (Waltham, MA, USA). Specific antibodies against collagen type I a 1 chain (COL1A1), runt-related transcription factor 2 (RUNX2), SP7, NQO-1 and nuclear matrix protein p84 (THOC1) were obtained from Abcam (Cambridge, UK). Specific antibodies against Nrf2, GAPDH, Phospho-Smad1 (Ser463/465)/Smad5 (Ser463/465)/Smad9 (Ser465/467), and Smad1 were purchased from Cell Signaling Technology (Danvers, MA, USA). Specific antibodies against Nrf2, heme oxygenase-1 (HO1) and β-actin were obtained from Proteintech (Wuhan, China). Specific antibodies against cleaved caspase-3 (CC3), BMP2, BAX and BCL2 were obtained from Beyotime Biotechnology (Shanghai, China).

### Cell proliferation assay

To evaluate the effects of Aucubin on the proliferation of hBM-MSCs, we inoculated the cells with a density of 5 × 10^3^ cells/well into 96-well plates. After 24 h adhering, different concentrations of Aucubin (0, 0.01, 0.1, 1 μM) were cultured for 1, 3, 5, and 7 days. Subsequently, the medium was replaced with 10% Cell Counting Kit-8 (CCK-8, Beyotime Biotechnology, Shanghai, China) in 100 μl low-sugar Dulbecco's modified Eagle's medium (L-DMEM) and incubated for 4 h at 37 °C. Finally, optical density was measured at 450 nm on a microplate reader (ELX808; BioTek, Winooski, VT, USA).

### Osteogenic differentiation assay

hBM-MSCs were cultured in a growth medium (Cyagen Biosciences, Guangzhou, China) in cell culture plates or flasks at a density of 3 × 10^4^/cm^2^ and incubated at 37 °C with 5% CO_2_. When cells reached at 80% confluence, the cells were incubated in osteogenic differentiation medium (ODM; low-sugar Dulbecco's modified Eagle’s medium; 10% fetal bovine serum, 100 nM dexamethasone, 10 mM β-glycerophosphate, 1% penicillin–streptomycin and 0.05 mM L-ascorbic acid-2-phosphate) with different concentrations of Aucubin (0, 0.01, 0.1, 1 μM). The medium was changed by every 3 days.

### Alkaline phosphatase (ALP) staining

hBM-MSCs were cultured in 12-well plates with an osteogenic differentiation medium for 3 days. We could perform ALP staining. The cells were firstly washed with PBS three times, fixed with 4% paraformaldehyde for 15 min and then washed again but with double-distilled water (ddH_2_O) every 3 min for three times. Finally, the cells were stained with the BCIP/NBT ALP color development kit (Beyotime Biotechnology, Shanghai, China). According to the manufacturer’s instructions, ALP activity was determined by the ALP activity assay (Beyotime Biotechnology, Shanghai, China). Then, the solutions were collected and measured by a microplate reader (ELX808; BioTek) at 405 nm.

### Alizarin Red staining

Mineral deposition was assessed by Alizarin Red staining (ARS) (Cyagen Biosciences, Guangzhou, China) after inducting osteogenic differentiation for 14 days. The cells were firstly washed with PBS three times, fixed with 4% paraformaldehyde for 15 min and then washed again but with double-distilled water (ddH_2_O) every 3 min for three times. Finally, the cells were stained with Alizarin Red S solution for 10–15 min at room temperature. To quantify the staining, mineralized deposits were dissolved in 10% cetylpyridinium chloride (Sigma, Shanghai, China). Afterward, the solutions were collected and measured by a microplate reader (ELX808; BioTek) at 560 nm.

### RNA isolation and quantitative RT-PCR

hBM-MSCs were differentiated in osteogenic differentiation medium adding AU (0, 0.01, 0.1, 1 μM) for 3 and 7 days. Total cellular RNA was extracted by RNAiso reagent (Takara, Kusatsu, Japan), and the absorbance of the solution at 260 nm was measured by NanoDrop 2000 (Thermo Fisher Scientific, MA, USA). The Double-Strand cDNA Synthesis Kit (Takara) was used to reverse-transcribe total RNA (≤ 1 μg) into cDNA in a reaction volume of 20 μl. SYBR Green PCR Master Mix reagent (Takara) was used to quantify cDNA (2 μl), which was then detected in triplicate by the StepOnePlus System (Applied Biosystems). Results were normalized to 18S as the housekeeping gene. All of the primers were synthesized by Sangon Biotech (Shanghai, China). The qRT-PCR reaction conditions are as follows: 95 °C for 30 s, followed by 40 cycles of 95 °C for 5 s, and 60 °C for 30 s. The expression levels of all of the genes were evaluated by 2^−△△Ct^ method. All primers used in this experiment were synthesized by Sangon Biotech, as shown in Table 1.

**Table1:** Sequences of primers for qRT-PCR

Gene	Forward primer (5'to3')	Reverse primer (5'to3')
COL1A1	CAGATCACGTCATCGCACAAC	GAGGGCCAAGACGAAGACATC
RUNX2	TGGTTACTGTCATGGCGGGTA	TCTCAGATCGTTGAACCTTGCTA
SP7	AGCCCATTAGTGCTTGTAAAGG	CCTCTGCGGGACTCAACAAC
ALP	ACCACCACGAGAGTGAACCA	CGTTGTCTGAGTACCAGTCCC
OCN	CCACCGAGACACCATGAGAG	CTGGACTCTGCACCGCTG
OPN	CTCCATTGACTCGAACGACTC	CAGGTCTGCGAAACTTCTTAGAT
18S	CCAGACAAATCGCTCCACCAAC	GACTCAACACGGGAAACCTCAC

### Western blotting analysis

hBM-MSCs were differentiated in osteogenic differentiation medium adding AU (0, 0.01, 0.1, 1 μM) for 3 and 7 days. With regard to inhibitor studies, cells were treated with AU (1 μM) and noggin (100 ng/mL) /LDN193189 (100 nM) for 3 days [22]. The above reagents were given again 2 h before sample collection. For studies on antioxidant stress injury of AU, 300 μM H_2_O_2_[12, 23] was added to an osteogenic differentiation medium to create an oxidative stress environment in vitro. After stimulating cells with 300 μM H_2_O_2_, the various concentrations of AU were added immediately. Cells were differentiated in an osteogenic differentiation medium for 3 days. AU and H_2_O_2_ were given again 24 h before sample collection and the subsequent experiment. The procedure of nuclear and cytoplasmic extraction was according to the manufacturer’s instructions. Subsequently, cells were lysed in RIPA buffer on ice with phosphatase inhibitors and protease inhibitors (Fdbio Science, Hangzhou, China) for 30 min, vortexing every 5 min. Centrifuge at 14,000 rpm at 4 °C for 10 min to obtain supernatant and then add 5X dual-color protein loading buffer (Fdbio Science, Hangzhou, China). After electrophoresis, the SDS polyacrylamide gel was transferred to a polyvinylidene fluoride (PVDF) membrane (MilliporeSigma). PVDF membranes were blocked in 10% skimmed milk with Tris-buffered saline-Tween (TBST) solution for 1 h at room temperature and then incubated with primary antibodies at 4 °C for at least 12 h. After washing with TBST for three times (5–10 min each) and incubating with horseradish peroxidase-conjugated secondary antibodies (Beyotime Biotechnology, Shanghai, China) for 1 h at room temperature, proteins were visualized by using an enhanced chemiluminescent detection reagent (MilliporeSigma) and an XRS chemiluminescence detection system (Bio-Rad Laboratories, Hercules, CA, USA). The densitometric analysis of the signal intensities was quantified using ImageJ 1.53a and normalized to GAPDH/β-actin/THOC1 as the housekeeping proteins and the corresponding unphosphorylated form of the protein of interest.

### Immunofluorescence assay

hBM-MSCs were differentiated in osteogenic differentiation medium adding AU (0, 0.01, 0.1, 1 μM) for 3 days. With regard to inhibitor studies, cells were treated with AU (1 μM) and noggin (100 ng/mL) /LDN193189 (100 nM) for 3 days. Pertaining to AU antioxidant injury studies, cells were treated with both 1 μM AU and 300 μM H_2_O_2_ for 3 days. The ODM adding AU and H_2_O_2_ was changed after 2 days of osteogenesis. Thereafter, cells were treated with 4% paraformaldehyde for 15 min at room temperature. After washing with PBS for three times (3 min each), the cells were incubated for 5 min with 0.1% TritonX-100 in PBS and blocked in 5% bovine serum albumin for 30 min. Treated cells were washed for three times and incubated with primary antibodies (anti-COL1A1 (1:200; Abcam), anti-RUNX2 (1:200; Abcam) and anti-Nrf2 (1:200; Proteintech)) at 4 °C for at least 12 h. Then the cells were incubated with fluorescence-conjugated secondary antibody for 1 h and stained with DAPI staining solution (Nanjing KeyGen Biotech, Nanjing, China). A fluorescent microscope (Leica Camera) was used to capture the images, and the fields were randomly selected.

### DCFH-DA staining

To investigate whether AU reduced ROS production caused by adding 300 μM H_2_O_2_ and plays a role of antioxidant stress injury, we constructed 3 groups: (1) NC group: without treatment. (2) H_2_O_2_ group: adding 300 μM H_2_O_2_ to ODM. (3) H_2_O_2_ + AU group: adding 300 μM H_2_O_2_ and 1 μM AU to ODM at the same time. The measurement procedures of reactive oxygen species (ROS) production were according to the manufacturer's instructions. After treating for 6 h, hBM-MSCs were incubated with DCFH-DA (Beyotime Biotechnology, Shanghai, China) for 20 min at 37 °C and then washed with L-DMEM three times. The images were immediately captured by a fluorescence microscope (Leica Camera). The mean fluorescence intensity was analyzed using the ImageJ software.

### In vivo experiment

This study was approved by the Institutional Animal Care and Use Committee of the Second Affiliated Hospital, School of Medicine, Zhejiang University (approval number: 2018–078). Eighteen male Sprague Dawley rats (8 weeks old, 200 g) were used to create tibial fracture models according to the requirements of the Animal Care and Use Committee of Zhejiang Province and the standards for the care and use of laboratory animals. These rats were divided equally into three groups of six rats each: (1) Blank group: without treatment. (2) PBS group: 20 μl PBS injection at the fracture site. (3) AU group: AU (20 μl Aucubin at 10 μM)) injection at the fracture site. All surgical procedures are performed by experienced orthopedic surgeons. Briefly, the rats were anesthetized with 0.3% sodium pentobarbital (Sigma, Shanghai, China) by intraperitoneal injection. The calves were shaved with a shaving machine and the skin was disinfected with an alcohol wipe. An incision was made in the proximal tibia and the tibia was broken under the tibial tuberosity after blunt dissection of the muscle. After wiping the knee joint with alcohol, a 1.3-mm intramedullary fixation pin was used for closed reduction of internal fixation of the tibial fracture site. All groups were operated on the left hind leg of the rats. The incision was then closed with absorbable sutures. Postoperatively, 20 μl Aucubin (10 μM)) was injected locally into the tibial fracture site of the rats in the AU group every 2 days, 20 μl PBS was injected locally into the tibial fracture site of the rats in the PBS group every 2 days and no treatment in the Blank group. After 6 weeks, all rats were executed and samples were fixed in 4% paraformaldehyde for subsequent experiments.

### Micro-CT and bone histomorphometric analysis

After samples have been fixed in water for 48 h, micro-CT (Scanco Medical, Brüttisellen, Switzerland) was used to scan and analyze the tibia. Parameters: isometric resolution of 14.8 μm, exposure time of 300 ms, and X-ray energy settings of 70 kV and 80 μA. Bone trabecular volume per total volume (BV/TV), bone trabecular surface per bone volume (BS/BV), mean bone trabecular thickness (Tb.Th), mean bone trabecular number (Tb.N) and mean bone trabecular separation (Tb.Sp) were used to quantitatively assess the microstructure of the tibia.

### Histological evaluation

After micro-CT, the samples were decalcified with 10% ethylenediaminetetraacetic acid (EDTA, Sigma) with 0.1 M PBS for 2 months, with mixed solutions changed per week. After decalcification, the samples were embedded in paraffin, serially sectioned at a thickness of 3 μm and placed on polylysine-coated slides, dewaxed and subjected to hematoxylin and eosin (H&E), Masson’s trichrome, Safranin O and fast green.

### Statistical analysis

Data are expressed as mean ± SD. All statistical analysis was performed using GraphPad Prism (version 9.0; GraphPad Software, San Diego, CA, USA). All of the experiments were performed ﻿no less than three times. Differences between the two groups were analyzed using a two-tailed Student's t test. For comparisons between multiple groups (≥ 3 groups), one-way analysis of variance (ANOVA) and Bonferroni post hoc tests were used. *p* < 0.05 was considered to be statistically significant.

## Results

### AU did not affect the viability and proliferation of hBM-MSCs

**Fig. 1:**
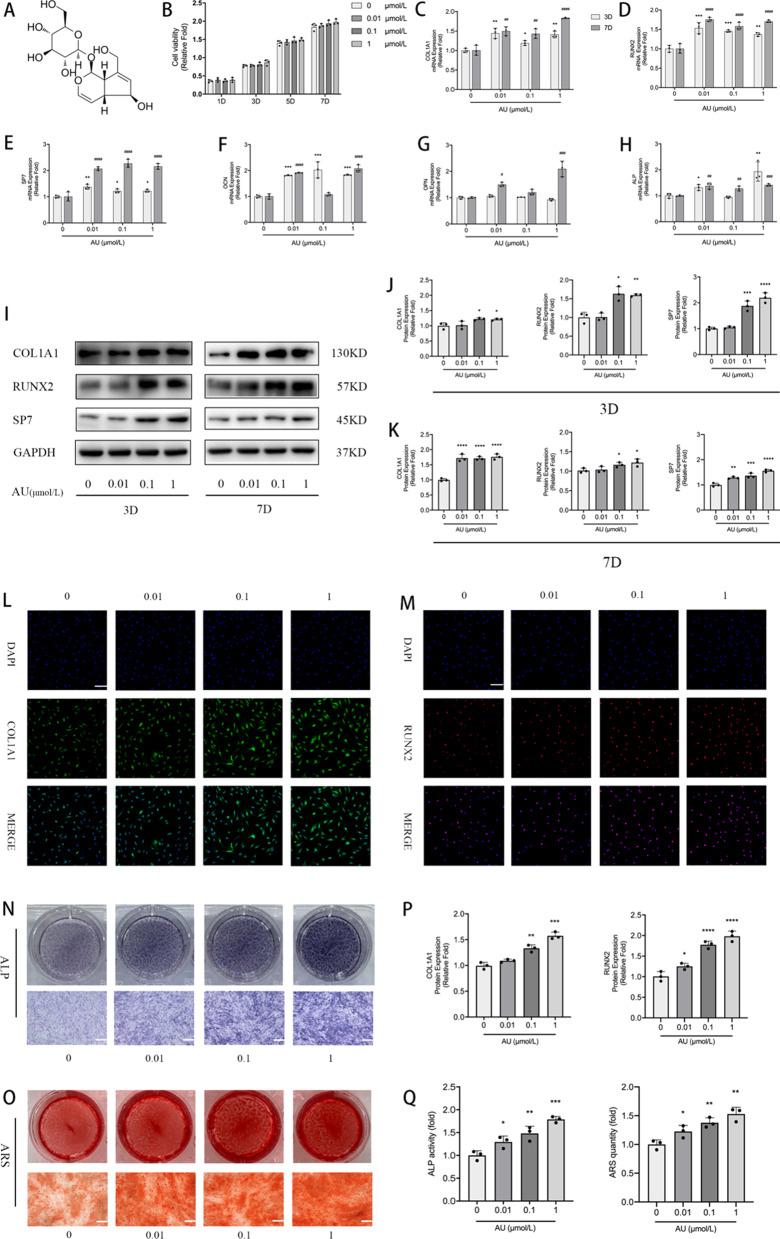
The effect of different concentrations of Aubucin on viability and ﻿osteogenic differentiation in hBM-MSCs. **a** The chemical structure of Aubucin. **b** The proliferation of hBM-MSCs was measured by CCK-8 assay after cell cultured with different concentrations of Aubucin (0–1 μM) for 1, 3, 5 or 7 days in hBM-MSCs growth medium. **c**-**h** The relative mRNA expression of osteogenesis-related genes (COL1A1, RUNX2, OCN, ALP, SP7 and OPN) was measured by qRT-PCR on days 3 and 7 after osteogenic differentiation. **i**-**k** The relative protein expression of osteogenesis-related genes (COL1A1, RUNX2 and SP7) were evaluated by western blotting analysis after ﻿osteogenesis for 3 and 7 days and normalized to GAPDH. **l**, **m**, **p** Immunofluorescence staining for COL1A1 and RUNX2 proteins after 3 days of osteogenesis. Scale bars, 50 μm. **n**, **o**, **q** ﻿Different concentrations of Aubucin (0–1 μM) promoted hBM-MSCs mineralization and ALP activity. ﻿ALP staining and activity was detected on day 3 of osteogenesis. ARS staining and quantitation was detected on day 14 of osteogenesis. Scale bars, 500 μm. All of the experiments were performed ﻿in triplicates. Data are expressed as mean ± SD. *P < 0.05, **P < 0.01, ***P < 0.001, ****P < 0.0001 compared to the control group on day 3. ^#^P < 0.05, ^##^P < 0.01, ^###^P < 0.001, ^####^P < 0.0001 compared to the control group on day 7

hBM-MSCs were cultured in hBM-MSC growth medium with different concentrations (0, 0.01, 0.1 and 1 μM) of AU for 1, 3, 5 and 7 days. The experimental results indicated that different concentrations of AU did not have a significant impact on the viability and proliferation of hBM-MSCs at different time nodes (Fig. 1b).

### AU promoted osteogenic differentiation of hBM-MSCs

To evaluate the role of AU in the osteoblastogenesis of hBM-MSCs, the mRNA and protein expression of bone-specific gene expression was examined by qRT-PCR, western blotting and immunofluorescence analysis. ALP staining and quantitative analysis were performed to evaluate ALP activity. ARS and quantitative analysis were performed to evaluate calcium deposition.

qRT-PCR analysis revealed that the mRNA expression of osteogenesis-related genes (COL1A1, RUNX2, SP7, OCN and ALP) was significantly increased on days 3 and 7 of osteogenesis compared to control group except the expression of OCN (day 7) and ALP (day 3) at 0.01 μM (Fig. 1c–f, h). OPN mRNA expression was statistically significant only at concentrations of 0.01 μM and 1 μM on day 7 of osteogenesis (Fig. 1g). Western blotting analysis indicated that the protein expression of osteogenesis-related genes (COL1A1, RUNX2 and SP7) was significantly increased at concentrations of 0.1 μM and 1 μM on day 3 of osteogenesis compared to control group, while no significant difference was observed at 0.01 μM (Fig. 1i, j). AU promoted the protein expression of COL1A1, RUNX2 and SP7 on day 7 of osteogenesis except the expression of RUNX2 at 0.01 μM (Fig. 1i, k). Similarly, immunofluorescence analysis suggested that AU notably increased the protein expression of COL1A1and RUNX2 on day 3 of osteogenesis compared to control group (Fig. 1l, m, p). We investigated the effect of AU on early mineralization and late calcium nodule formation during osteogenic differentiation by ALP and ARS, respectively. The results revealed that AU increased ALP activity on day 3 of osteogenesis and calcium deposition on day 14 of osteogenesis (Fig. 1n, o, q).

### AU-enhanced osteogenic differentiation of hBM-MSCs partly via the BMP2/Smads signaling pathway

**Fig. 2:**
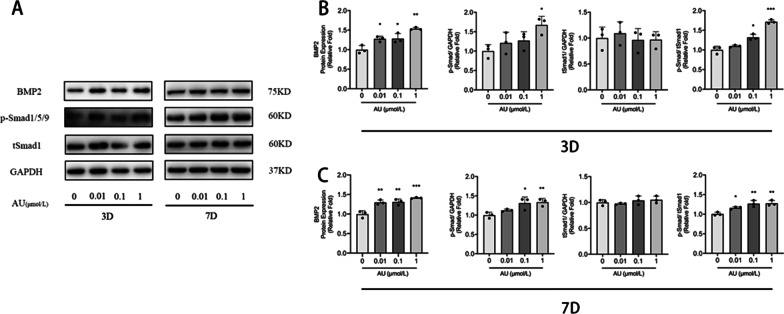
Aubucin promotes osteogenic differentiation of hBM-MSCs partly via the BMP2/Smads signaling pathway. **a**-**c** The relative protein expression of components of different signaling pathways (BMP2 and p-Smad1/5/9) was evaluated by western blotting analysis after osteogenesis for 3 and 7 days and normalized to GAPDH and tSmad1. All of the experiments were performed in triplicates. Data are expressed as mean ± SD. *P < 0.05, **P < 0.01, ***P < 0.001, ****P < 0.0001 compared to the control group

To investigate the mechanisms by which AU promotes osteogenic differentiation of hBM-MSCs, we applied western blotting analysis to detect the BMP2/Smads signaling pathway, which was essential in the osteogenic process. Western blotting indicated that increased protein expression of BMP2 was observed in the different concentrations of AU treatment groups on days 3 and 7 of osteogenesis. Similarly, the ratio of phospho-Smad1/5/9 and total Smad also significantly increased after treatment of AU (Fig. 2a–c). The results suggested that the BMP2/Smads pathway may be one of the major ways in which AU promoted osteogenic differentiation of hBM-MSCs.

### Blockade of BMP2 signaling by LDN193189 and noggin reversed AU-mediated effects in hBM-MSCs

**Fig. 3:**
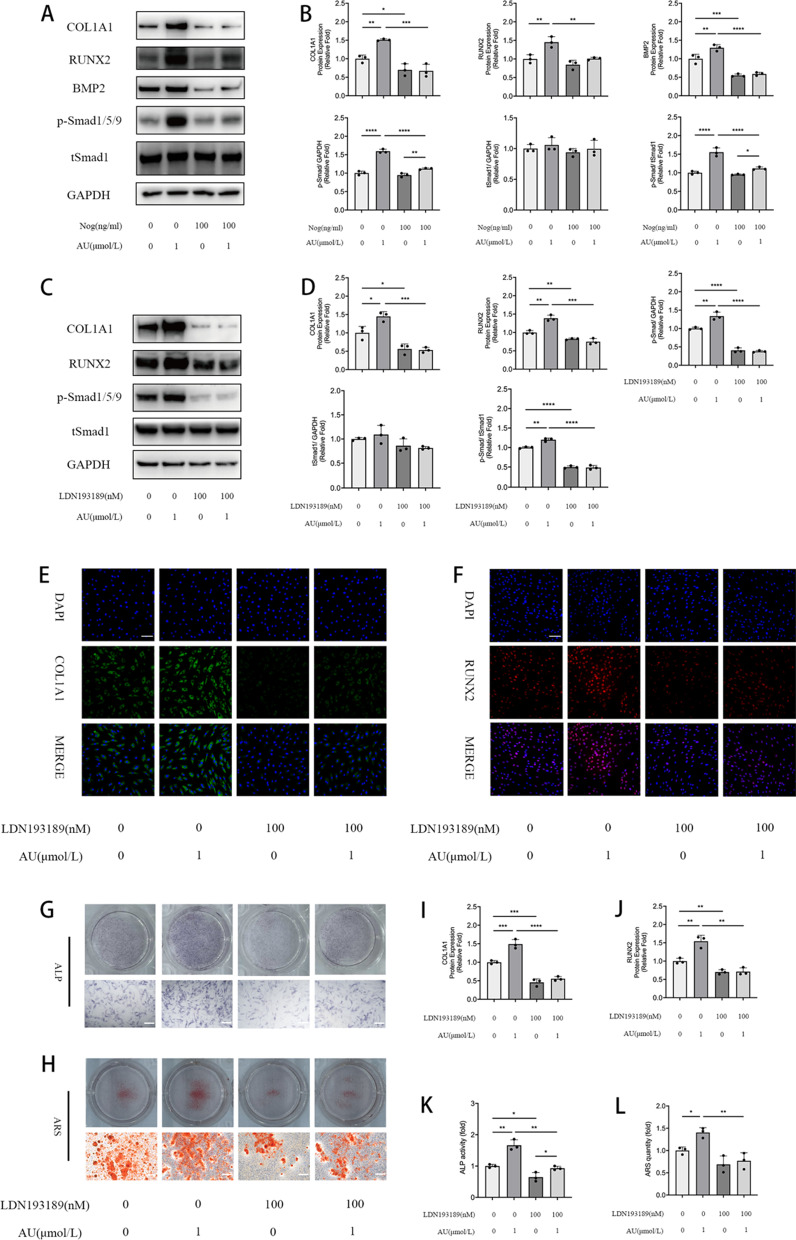
AU-mediated osteogenic response is inhibited by noggin (BMP antagonist) and LDN193189 (type I BMP receptor inhibitor). Confirming canonical BMP signaling blockade. **a**, **b** Phospho-Smad1/5/9, BMP2, COL1A1 and RUNX2 were quantified in hBM-MSCs after 3 days of treatment with AU and/or noggin by western blot analysis and normalized to GAPDH and tSmad1. **c**, **d** Phospho-Smad1/5/9, COL1A1 and RUNX2 were quantified in hBM-MSCs after 3 days of treatment with AU and/or LDN193189 by western blot analysis and normalized to GAPDH and tSmad1. **e**, **f**, **i**, **j** Representative images and quantification of immunofluorescence staining of COL1A1 and RUNX2. Scale bars, 100 μm. **g**, **h** Representative images of ALP and ARS. **k**, **l** Quantification of ALP and ARS. Scale bars, 500 μm. All of the experiments were performed in triplicates. Data are expressed as mean ± SD. Statistical significance is denoted in the graphs. *: P < 0.05; **: P < 0.01; ***: P < 0.001; ****: P < 0.0001

To investigate whether AU-enhanced osteoblastogenesis is mediated by BMP2 signaling, we inhibited its activation by specifically blocking BMPR type I members ALK2 and ALK3 using LDN193189 and segregating BMP2 using noggin. AU-induced expression of BMP2 signaling target genes BMP2 and p-Smad1/5/9 as well as of osteogenic markers COL1A1 and RUNX2 was downregulated after 3 days of BMP2 blockade, which can be measured by western blotting and immunofluorescence analysis (Fig. 3a–f, i, j). ALP and ARS staining indicated that AU-promoted ALP activity and mineralization were significantly attenuated by treating with LDN193189 and noggin (Fig. 3g, h, k, l). Thus, BMP2 signaling did mediate the effects of AU on the osteogenesis of hBM-MSCs.

### AU reversed oxidative damage and osteogenesis inhibition caused by exposure to H_*2*_*O*_*2*_

**Fig. 4:**
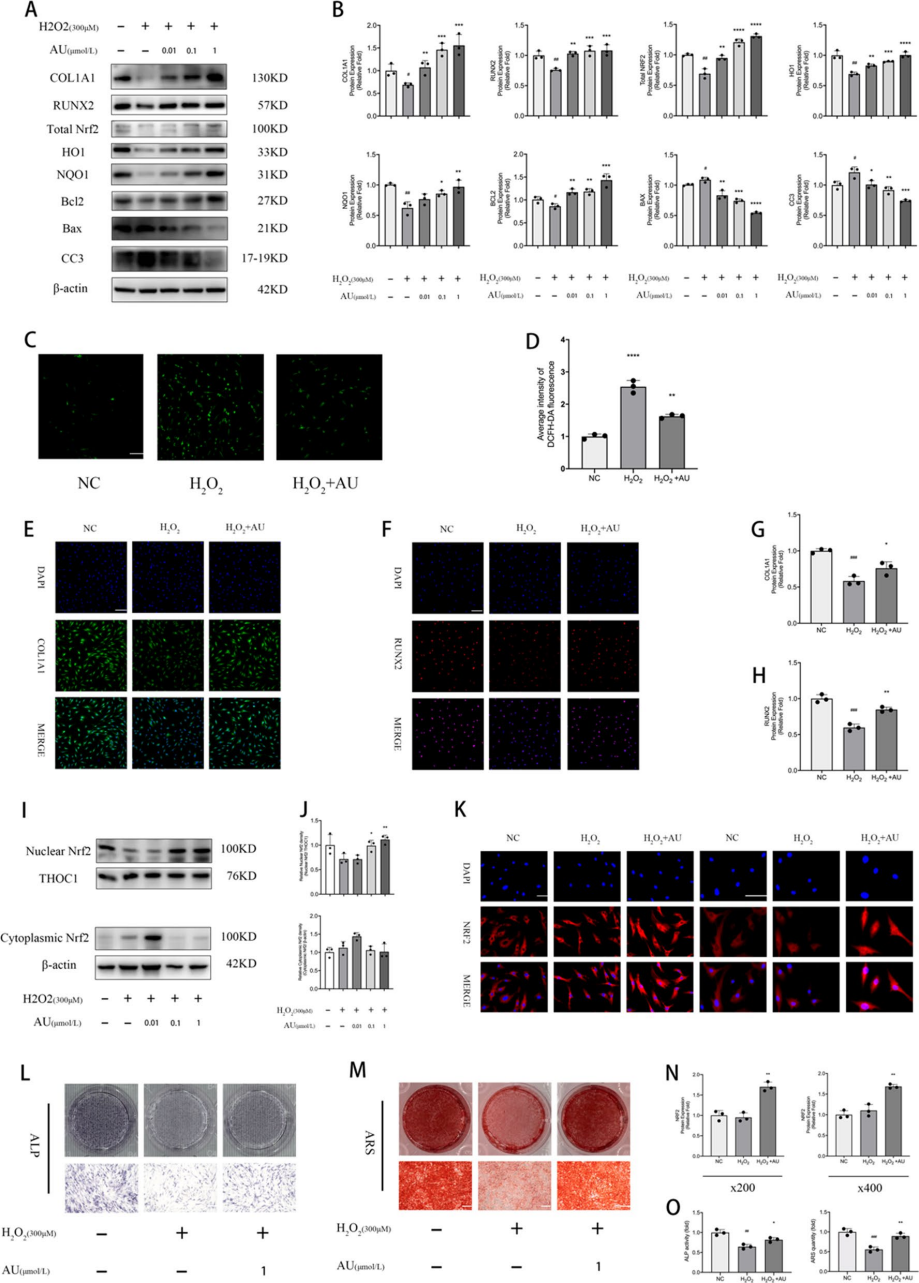
Aubucin reverses oxidative damage and osteogenesis inhibition caused by exposure to ﻿300 μM H_2_O_2_. **a**, **b** The relative protein expression of osteogenesis-related genes (COL1A1 and RUNX2), components of different signaling pathways (Total NRF2, HO1 and NQO1) and apoptosis-related genes (Bcl2, BAX and CC3) were evaluated by western blotting analysis after ﻿osteogenesis for 3 days and normalized to β-actin. **c**, **d** Representative micrographs and quantification of DCFH-DA staining of control group, H_2_O_2_ group, and H_2_O_2_ + AU (1 μM) group. Scale bars, 100 μm. **e**–**h** Expression of COL1A1 and RUNX2 proteins was determined by immunofluorescence on day 3 of osteogenesis. Scale bars, 50 μm. **i**, **j** Nuclear Nrf2 and cytoplasmic Nrf2 were quantified in hBM-MSCs after 3 days of treatment with AU and/or H_2_O_2_ by western blot analysis and normalized to THOC1 and β-actin. **k**, **n** Expression of Nrf2 protein was determined by immunofluorescence on day 3 of osteogenesis. Scale bars, 100 μm. **l**, **m**, **o** Aubucin (1 μM) promoted hBM-MSCs mineralization and ALP activity exposed to 300 μM H_2_O_2_. ALP staining and activity was detected on day 3 of osteogenesis. ARS staining and quantitation was detected on day 14 of osteogenesis. Scale bars, 500 μm. All of the experiments were performed in triplicates. Data are expressed as mean ± SD. ^*^P < 0.05, **P < 0.01, ***P < 0.001, ****P < 0.0001 compared to the H_2_O_2_ group. ^#^P < 0.05, ^##^P < 0.01, ^###^P < 0.001 compared to the control (NC) group

To determine the antioxidant effect of AU, an oxidative stress model in vitro was constructed by adding 300 μM of H_2_O_2_ to the osteogenic differentiation medium. After stimulating cells with 300 μM H_2_O_2_, the various concentrations of AU were added immediately. AU-induced expression of osteogenesis-related genes (COL1A1 and RUNX2) as well as of Nrf2 signaling target genes (total Nrf2, HO1 and NQO1) and of anti-apoptotic gene Bcl2 was significantly upregulated relative to H_2_O_2_ group after 3 days of AU and H_2_O_2_ treatment, while the expression of apoptotic genes BAX and CC3 was downregulated (Fig. 4a, b). DCFH-DA staining indicated that H_2_O_2_ significantly increased ROS levels compared to control group, and the treatment of AU (1 μM) partly reversed the effect (Fig. 4c, d). In consistent with the results of western blotting, immunofluorescence staining indicated that AU (1 μM) reversed the reduced protein expression of COL1A1 and RUNX2 exposed to H_2_O_2_ (Fig. 4e–h)_._ In addition, AU (1 μM) treatment promoted translocation of Nrf2 from cytoplasm to nucleus in the microenvironment of the exposed to H_2_O_2_ (Fig. 4i–k, n). The attenuated mineralization due to oxidative damage was reversed by the addition of AU (1 μM) as determined by ALP staining assay and ARS solution (Fig. 4l, m, o).

### AU accelerated bone-fracture healing in a rat tibial fracture model

**Fig. 5:**
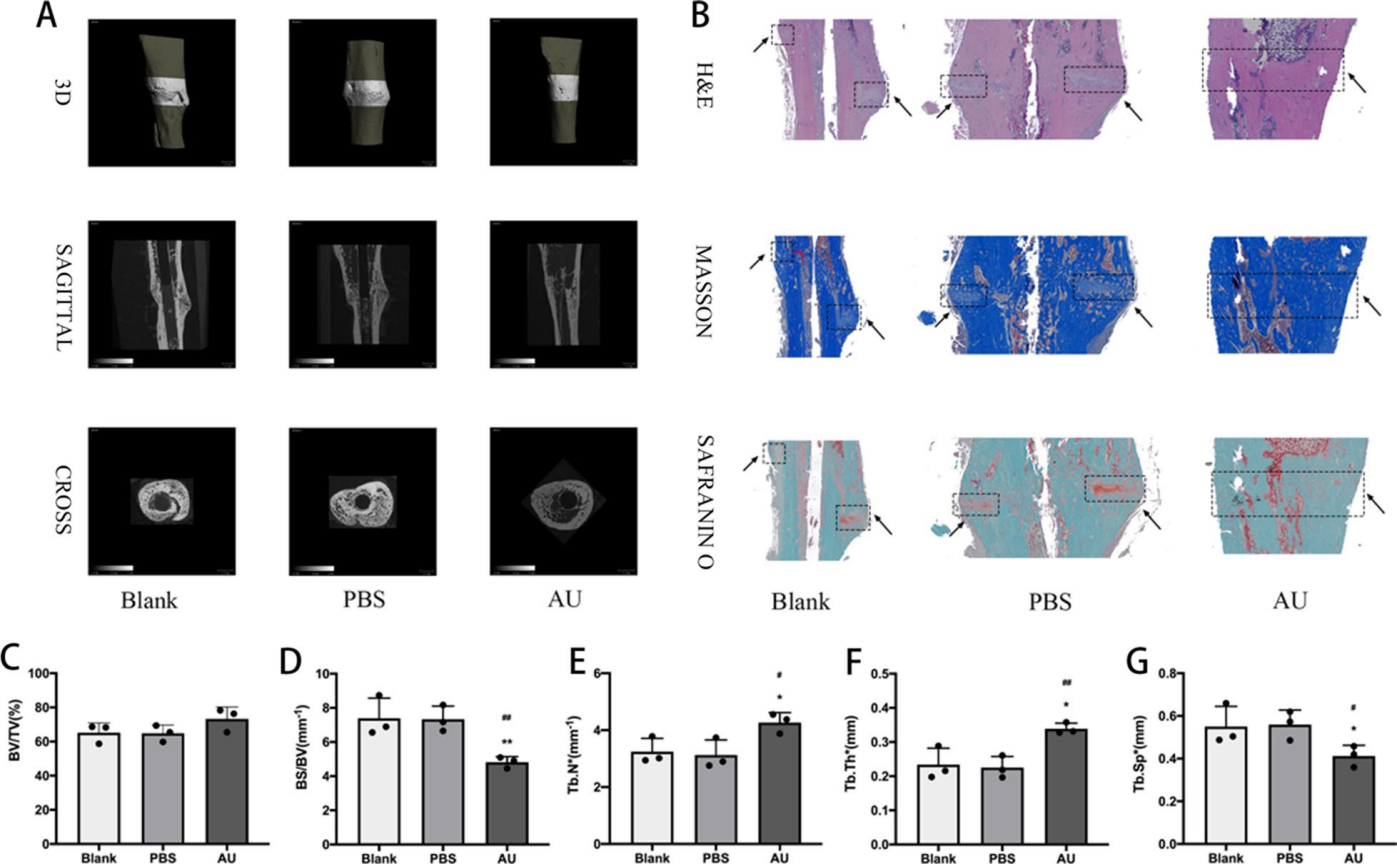
Aubucin promotes bone recovery in a rat tibial fracture model. **a** Micro-CT analysis for bone healing. **b** Histological analysis for bone healing. H&E, hematoxylin and eosin staining. Masson, Masson’s trichrome staining. Safranin O, Safranin O and fast green. **c**-**g** Specific indicators of bone healing (BV/TV, BS/BV, Tb.Th, Tb.N and Tb.Sp) are presented. Scale bars, 1 mm. All of the experiments were performed ﻿in triplicates. Data are expressed as mean ± SD. *P < 0.05, **P < 0.01 compared to the ﻿Blank group. ^#^P < 0.05, ^##^P < 0.01 compared to the PBS group

To further verify the effects of AU on bone-fracture regeneration and repair in vivo, we constructed a rat tibial fracture model. After 6 weeks, Micro-CT indicated that AU significantly accelerated bone-fracture healing. Fracture line disappeared and more bridging callus formation was presented in the fracture area compared to Blank and PBS group (Fig. 5a). In agreement with the results of micro-CT, histological evaluation (including H&E, Masson’s trichrome, Safranin O and fast green staining) showed that the AU group had better fracture union and cortex callus formation in contrast with Blank group and PBS group (Fig. 5b). Quantitatively, indicators of microstructural evaluation of bone (including BS/BV, Tb.Th, Tb.N, Tb.Sp) evidently increased in the AU group relative to Blank group and PBS group (Fig. 5d–g). However, BV/TV did not present a statistically significant difference (Fig. 5c).

## Discussion

Bone-fracture healing is a complex dynamic process, mainly dominated by mechanical stability and the microenvironment around the fracture region [5]. The local fracture microenvironment at a fracture site is rich in multiple cells, cytokines, growth factors, and ROS, which are essential for bone and soft tissue repair [5, 24]. When the homeostasis of the fracture microenvironment is disrupted, for instance, oxidative stress caused by excessive accumulation of ROS, bone-fracture healing will delay and even cause bone nonunion [1, 2]. It is reported that fractures locally and damaged soft tissues around produce a remarkable yield of ROS [9, 10]. Meanwhile, damage to the bone drilling during surgery and the nature of the internal fixation plate can further exacerbate the accumulation of ROS [12], leading to oxidative stress injury and then a failure of bone-fracture healing. Thus, it is crucial for bone-fracture healing to alleviate oxidative stress by inhibiting ROS. In this study, we first demonstrated the dual effects of AU in bone-fracture healing. AU could not only protect bone-fracture healing from oxidative stress injury via activation of Nrf2/HO1 signaling pathway but also improve bone-fracture healing by promoting osteogenesis of hBM-MSCs partly via BMP2/Smads signaling pathway.

AU was a small ﻿molecular compound derived from natural herbal medicines with low price and clinical application potential [18]. It has been reported that AU has a variety of pharmacological activities, including antioxidant, anti-aging, anti-inflammatory, antimicrobial and osteoprotective effects [18, 19]. Recently, research on osteoprotective effects of AU mostly focused on anti-osteoporosis and anti-apoptosis. Li et al. reported that Aucubin effectively promoted the differentiation of MG63 cells and exhibited anti-osteoporotic effects in a mouse model of osteoporosis induced by dexamethasone (Dex) or hydrogen peroxide [21]. Zhang et al. reported that Aucubin slowed the development of osteoporosis by inhibiting osteoclast differentiation through the Nrf2 pathway [20]. However, few studies mentioned the relationship between AU treatment and bone-fracture healing. In the present study, we revealed that AU at concentrations from 0.01 to 1 μM neither promoted nor inhibited the viability and proliferation of hBM-MSCs. Increased mRNA and protein expression of COL1A1 and RUNX2 after the treatment of different concentrations of AU were examined by qRT-PCR, western blotting and immunofluorescence analysis. AU promoted early and late mineralization in a dose-dependent manner based on ALP and ARS staining, respectively. These results showed that AU promoted osteogenic differentiation of hBM-MSCs. Furthermore, AU accelerated bone-fracture healing in a rat tibial fracture model in vivo.

BMP signaling was proved to have a fundamental role in both skeletal development and bone homeostasis. Type I and type II BMP receptors were involved in BMP signaling. When homomeric dimers of type II receptors bind to BMP ligands, they created a tetrameric complex with homomeric dimers of type I receptors, causing transphosphorylation of the type I receptors. This dynamic interaction resulted in the activation of Smads or MAPKs, which promoted the transcription of osteogenesis-related genes [25–27]. BMP2, which acted as a member of BMP ligands, exhibits high osteogenic activity [28]. BMP2 dramatically boosted osteocalcin expression, and short-term BMP2 expression was sufficient to irreversibly trigger bone and cartilage formation [29, 30]. MSC-specific *BMP2* DKO mice exhibited severe osteogenesis impairment and limb deformity [31, 32]. Chondrocyte-specific *BMP2* CKO mice suggested severe chondrocytes disorganization within the growth plate region [33]. In addition, studies showed that BMP2 activity was necessary for the initiation of bone-fracture healing. Although MSCs presented at the repair sites, these cells remain undifferentiated and stationary in the absence of BMP2 [27, 29, 32]. In our study, western blotting showed that protein expression of endogenous BMP2 and the ratio of phospho-Smad1/5/9 and total Smad after AU treatment during osteogenesis were significantly increased. To test whether BMP2 signaling activation mediates AU acts on osteoblastogenesis in vitro, we blocked BMP2 signaling with noggin (BMP antagonist) and LDN193189 (type I BMP receptor inhibitor). Interestingly, both treatments were sufficient to abrogate AU-enhanced BMP2/Smads signaling activation and osteoblast activity. These observations indicated that AU promoted the osteogenic differentiation of hBM-MSCs partly by modulating the canonical BMP2/Smads pathway.

Excessive ROS, caused by bone-fracture and internal fixation surgery, leads to oxidative stress [9, 10]. Oxidative stress was one of the major reasons for osteoblast dysfunction and apoptosis by damaging cell structure, nucleic acids and proteins [8]. Finally, the failure of osteogenesis of BM-MSCs led to bone nonunion [8, 34]. Thus, anti-oxidative stress was essential for bone-fracture healing. It was reported that Nrf2-mediated signaling played a major role in anti-oxidative damage. Once stimulated by oxidative insult, Nrf2 dissociated from Keap1 and translocated from cytoplasm to nucleus, binding to the antioxidant response element (ARE) in the promoter of antioxidant genes and then activating the expression of antioxidant enzymes such as HO1 and NQO1 [21, 35]. In our study, we did find the AU promoted translocation of cytoplasmic Nrf2 to the nucleus and increased protein expression of HO1 and NQO1 in vitro. Increased protein expression of COL1A1 and RUNX2 measured by western blotting and immunofluorescence indicated AU reversed osteogenesis inhibition caused by oxidative stress. ALP staining and APS also validated this result. Overall, these results suggested that AU reversed oxidative damage and osteogenesis inhibition via the Nrf2-mediated signaling pathway.

Our study had some limitations. Firstly, we demonstrated that AU promoted osteogenic differentiation of hBM-MSCs partly via the canonical BMP2/Smads pathway. However, whether non-Smad-dependent BMP2 signaling was involved in the regulation of osteogenic differentiation of hBM-MSCs by AU treatment still needs further investigation. Secondly, other signaling pathways related to osteogenesis, such as Wnt/β-catenin, NF-κβ, ERK/MAPK and PI3K-AKT pathways whether they were activated or not, have not been elucidated. Thirdly, we were unable to obtain quantitative or qualitative changes in ROS levels around tibial fractures in rats after AU treatment.

## Conclusion

**Fig. 6:**
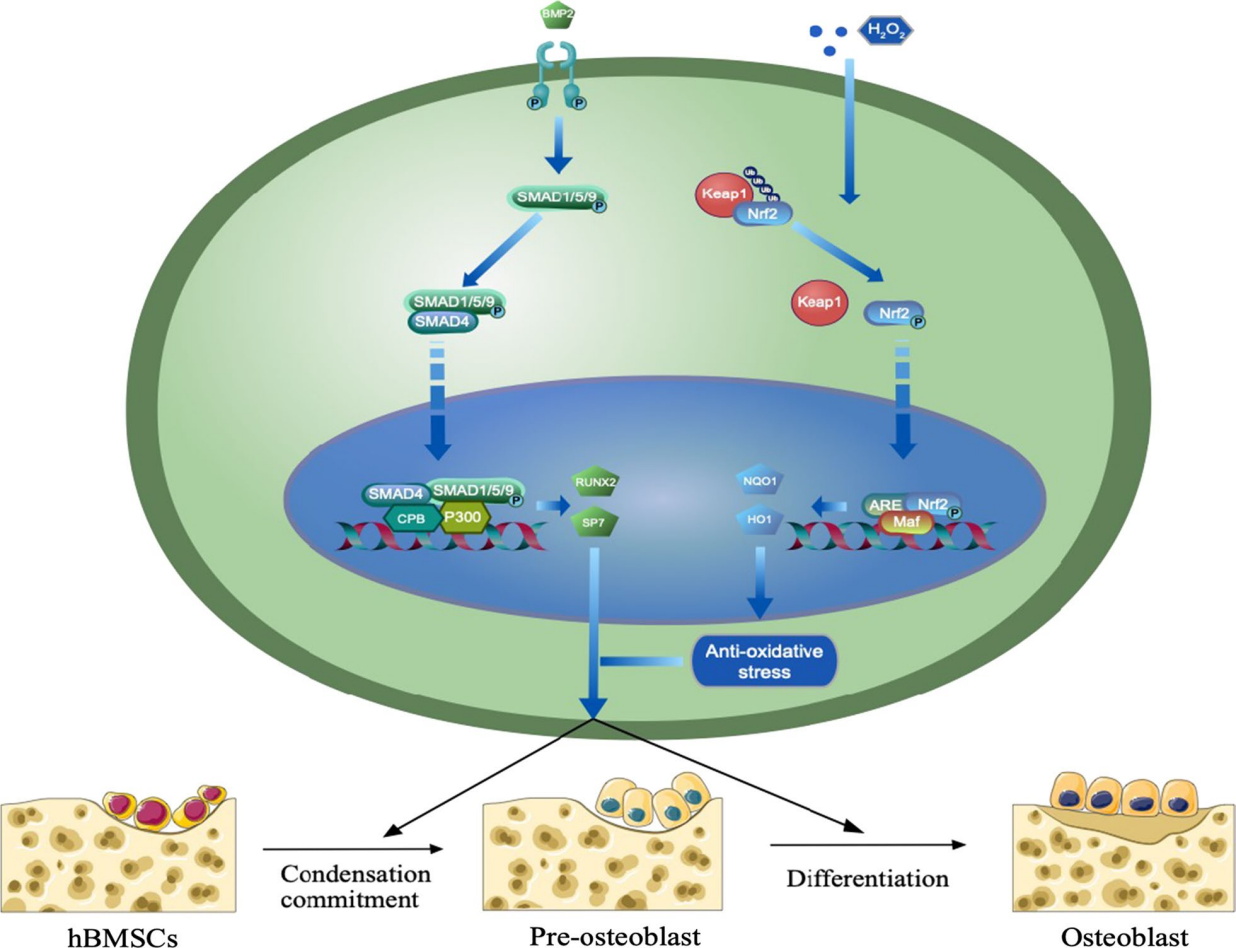
Schematic of the experiment

In conclusion, our study demonstrates the dual effects of AU in not only promoting bone-fracture healing by regulating osteogenesis of hBM-MSCs partly via canonical BMP2/Smads signaling pathway but also suppressing oxidative stress damage via partly Nrf2/HO1 signaling pathway (Fig. 6).

## Data Availability

The datasets used and/or analyzed during the current study are available from the corresponding author on reasonable request.

## Abbreviations

AU: Aucubin
hBM-MSCs: Human bone marrow-derived mesenchymal stromal cells
BM-MSCs: Bone marrow-derived mesenchymal stromal cells
BMP2: Bone morphogenetic protein 2
Nrf2: Nuclear factor erythroid-2-related factor 2
HO1: Heme oxygenase-1
ROS: Reactive oxygen species
COL1A1: Collagen type I a 1 chain
RUNX2: Runt-related transcription factor 2
OCN: Osteocalcin
OPN: Osteopontin
Bcl2: B cell lymphoma 2
Bax: Bcl2-associated X protein
CC3: Cleaved caspase-3
CCK-8: Cell Counting Kit-8
ALP: Alkaline phosphatase
ARS: Alizarin red staining
qRT-PCR: Quantitative RT-PCR
PVDF: Polyvinylidene fluoride
TBST: Tris-buffered saline-Tween
BV/TV: Bone trabecular volume per total volume
BS/BV: Bone trabecular surface per bone volume
Tb.Th: Mean bone trabecular thickness
Tb.N: Mean bone trabecular number
Tb.Sp: Mean bone trabecular separation
